# Molecular and immunological characteristics of postoperative relapse in lymph node‐positive esophageal squamous cell cancer

**DOI:** 10.1002/cam4.7228

**Published:** 2024-05-11

**Authors:** Hua‐guang Pan, Han‐lin Fang, Chan Zhu, Si Li, Huan Yi, Xing Zhang, Xiang‐yu Yin, Yun‐jie Song, Dongsheng Chen, Chun‐tong Yin

**Affiliations:** ^1^ Department of Thoracic Surgery The First Affiliated Hospital of Anhui Medical University Hefei Anhui China; ^2^ Jiangsu Simcere Diagnostics Co., Ltd., Nanjing Simcere Medical Laboratory Science Co., Ltd. The State Key Lab of Translational Medicine and Innovative Drug Development Nanjing China; ^3^ Department of Biological Sciences Xi'an Jiaotong‐Liverpool University Suzhou China

**Keywords:** esophageal squamous cell carcinoma, genomic landscape, immune infiltration, lymph nodes, *MUC16*, primary tumor

## Abstract

**Background:**

The molecular and immunological characteristics of primary tumors and positive lymph nodes in esophageal squamous cell carcinoma (ESCC) are unknown and the relationship with recurrence is unclear, which this study attempted to explore.

**Methods:**

A total of 30 ESCC patients with lymph node positive (IIB‐IVA) were enrolled. Among them, primary tumor and lymph node specimens were collected from each patient, and subjected to 551‐tumor‐targeted DNA sequencing and 289‐immuno‐oncology RNA panel sequencing to identify the different molecular basis and immunological features, respectively.

**Results:**

The primary tumors exhibited a higher mutation burden than lymph nodes (*p* < 0.001). One‐year recurrent ESCC exhibited a higher *Mucin16 (MUC16)* mutation rate (*p* = 0.038), as well as univariate and multivariate analysis revealed that *MUC16* mutation is independent genetic factor associated with reduced relapse‐free survival (univariate, HR: 5.39, 95% CI: 1.67–17.4, *p* = 0.005; multivariate, HR: 7.36, 95% CI: 1.79–30.23, *p* = 0.006). Transcriptomic results showed non‐relapse group had higher cytolytic activity (CYT) score (*p* = 0.025), and was enriched in the IFN‐α pathway (*p* = 0.036), while those in the relapsed group were enriched in the TNF‐α/NF‐κB (*p* = 0.001) and PI3K/Akt pathway (*p* = 0.014).

**Conclusion:**

The difference in molecular characteristics between primary lesions and lymph nodes may be the cause of the inconsistent clinical outcomes. Mutations of *MUC16* and poor immune infiltration are associated with rapid relapse of nodes‐positive ESCC.

## Graphical abstract

Difference of molecular characteristics between primary lesions and lymph nodes may lead to their inconsistent clinical responses, 1‐year non‐relapse patients have better characteristics of immune infiltration, Mutations of MUC16 is associated with rapid relapse of nodes‐positive ESCC.

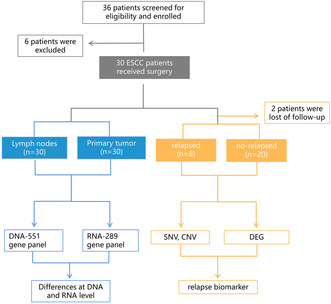

## 1 INTRODUCTION

Esophageal cancer (EC) is one of the deadliest cancers in the world, ranking 8th in incidence and 6th in mortality among all types of cancer in the world.^1^ Esophageal squamous cell carcinoma (ESCC) is the most common subtype of EC in China. The most common and effective treatment for early stage ESCC is surgical resection. Most patients have locally advanced EC when first diagnosed, with poor prognosis and high fatality rate, and surgical therapy alone is typically insufficient.^2^ Nowadays, preoperative chemoradiation or perioperative chemotherapy is utilized as a surgical adjunct.^3^ However, patients may still develop distant metastases or local recurrence after radical treatment.

Lymph node is a major hub for the growth of metastatic tumor cells, and lymph node metastasis (LNM) has been shown to be an important prognostic indicator for ESCC.^4^ Some ESCC patients have been found to respond inconsistently to treatment between primary and LNM, which may be attributed to intrinsic differences in different tissues.^5^, ^6^ Otto et al.^7^ compared tumor regional lymph nodes and distal lymph nodes in patients with different stages of EC and found that the more advanced the tumor, the more immunosuppressive the lymph nodes before metastasis showed. When the lymph node microenvironment is one in which immune function is evaded or suppressed, malignant tumors are more likely to migrate, grow, and respond poorly to treatment. In addition to differences in response to therapy, the characteristics of the primary tumor and LNM also differ in terms of patient prognosis. In a study that included 44 ESCC patients with lymph node metastases, after neoadjuvant chemotherapy, there was no significant correlation between PD‐L1 positivity in the primary tumor and prognosis (*p* = 0.31), whereas pathologic lymph node metastases and positivity for PD‐L1 in lymph nodes were independent poor prognostic factors for patients (*p* = 0.0122, *p* = 0.0463).^8^

In contrast, differences in the immune microenvironment between primary and lymph node metastases without intervention are unclear, and data supporting this view in the Chinese population are lacking. Currently, there are few studies of molecular characteristics of tumor cells between primary and lymph node metastases in patients with ESCC. Some studies have reported differences in mutation frequencies between the two, but these studies had small sample sizes and did not analyze the relationship between mutation and prognosis.^9^, ^10^ In this study, we focused on patients with ESCC and aimed to analyze the differences in molecular characteristics between primary foci and lymph node metastases of ESCC, including gene mutation and tumor immune microenvironment, and to explore biomarkers associated with prognosis. We hope that our findings will help to elucidate some pathological mechanisms of EC and further facilitate the diagnosis and treatment of ESCC.

## 2 METHODS

### 2.1 Study design and participants

The Ethics Committee of First Affiliated Hospital of Anhui Medical University has given its approval (PJ2022‐11‐13) for this retrospective study in accordance with the ethical principles stated by the Declaration of Helsinki, and all patients have signed written informed consent. Experimental subjects were not randomized into groups, and experimenters and patients were not blinded, as our study was a pilot exploratory study. The following were the qualifying requirements: (1) had postoperative pathological examination confirmed ESCC with LNM; (2) had undergone McKeown minimally invasive esophagectomy; (3) had total mediastinal and abdominal lymph node dissection (extended 2‐field dissection); (4) had postoperative pathological examination confirmed ESCC with LNM; (5) had the surgery was carried out by the same medical team, which included surgeons experienced in performing over 800 MIEs. These were the conditions for exclusion: (1) underwent palliative resection; (2) records were incompleted; (3) received neoadjuvant therapy.

All enrolled patients received postoperative adjuvant chemoradiotherapy according to NCCN guidelines. CT images of the chest and abdomen‐pelvis were performed 3 months after surgery to look for recurrence. Disease occuring at the anastomosis or LNM dissection was known as local recurrence. Recurrence in an area other than the surgical site, such as the lung, brain, liver or bone, was referred to as distant recurrence.^11^ If appropriate, tissue biopsy would be used to verify the recurrence.

### 2.2 Sample collection and preparation

All esophageal and regional lymph nodes in this study were surgical specimens. Following their fixation in 10% formalin, the samples were embedded in paraffin wax and sliced into 4‐μm slices. All tissues obtained were stained with hematoxylin–eosin. Two senior pathologists examined each sample, made a pathological diagnosis and estimated the percentage of tumor cells in each sample. To ensure that the purity of the tumor met the requirements of sequencing, the percentage of tumor cells in primary tumor was ≥20%, and the percentage of tumor cells in lymph node was ≥5%.

### 2.3 DNA extraction and library preparation

DNA sequencing was performed on clinically collected formalin‐fixed paraffin‐embedded (FFPE) tissues. The Genomic DNA Tissue Extraction Kit (Concert®) was used to extract the genomic DNA (gDNA). Quality assessment and concentration detection of DNA samples were measured using Qubit 3.0 with a dsDNA HS Assay Kit (Life Technologies) and the quality control of gDNA was performed by the 4200 Agilent TapeStation (Agilent). After that, an enzymatic fragmentation kit was used to shear 1 μg of gDNA into fragments (200–300 bp). These fragments were then subjected to end‐repairing, A‐tailing, and adaptor ligation using reagents from the KAPA Hyper DNA Library Prep kit (Roche Diagnostics) in that order. Size selection was then carried by removing the unligated adaptors using Agencourt AMPure XP beads (Beckman Coulter), and the linked products were amplified by PCR to form a pre‐library for hybridization.

### 2.4 Library sequencing and mutation analysis

In order to achieve target enrichment, indexed DNA libraries were combined and hybridized with specially made biotinylated‐DNA probes (Jiangsu Simcere Diagnostics Co., Ltd.) that were intended to target 551 genes associated with cancer. As directed by the manufacturer, enriched libraries were amplified and sequenced on Illumina NovaSeq 6000 platforms (Illumina, San Diego, CA), by which 150 bp paired‐end reads were generated. The fastp software (v.2.20.0) was used to trim the adapters and filter the low‐quality bases.^12^ The BWA‐MEM (v.0.7.17) algorithm was applied to align the reads to the reference genome (UCSC hg19/GRCh37).^13^ Duplicate reads from PCR were excluded using Dedup with Error Correct.

Single nucleotide variations (SNVs) and insertions/deletions (Indels) were called and annotated by VarDict (v.1.5.7) and InterVar, respectively.^14^ Afterwards, the variants were filtered for common single nucleotide polymorphisms (SNPs) through several databases, including 1000 Genome Project (Aug 2015) and Exome Aggregation Consortium (ExAC) Browser28 (v.0.3). CNV kit (dx1.1) was used to identify copy number variations (CNVs), which involved amplification and deletion.^15^ Only insertions and deletions (Indels) and single nucleotide variations (SNVs) in the coding area were taken into account in TMB assessments. MSI status only selected sequence depth greater than 50. The analysis of SNVs and CNVs for all samples was visualized using the R package “maftools”, with the top mutated genes and the corresponding mutation types and mutation frequencies are clearly visible.^16^

### 2.5 Transcriptional profiling

In order to perform RNA‐seq, total RNA from FFPE samples was extracted using the QIAGEN RNeasy FFPE Kit and it was then hybridized to a code set for the NanoString PanCancer 289‐immuno‐gene panel, through which the genes can be quantified on the nCounter platform (NanoString Technologies, Seattle, WA). This panel allows concurrent detection of 289 immune‐related genes including housekeeping genes (Table S1). As advised by the manufacturer, housekeeping genes were utilized to normalize the expression data using the nSolver 2.6 program. According to the manufacturer's specification, the panel includes 56 marker genes for 14 types of immune cell (mast cells, T cells, CD8 T cells, B cells, exhausted CD8 cells, NK‐CD56 cells, NK cells, dendritic cells, macrophages, cytotoxic cells, Th1 cells, CD45 cells, and Treg cells) (Table S2). The geometric mean expression levels of the constituent genes were used to compute the metagene scores.^17^ Additionally, 8 TiME signatures (cytotoxic T lymphocyte levels, chemokines, total tumor‐infiltrating lymphocyte score, T‐effector score, T cell markers, gene expression profiling score, cytolytic activity score, and interferon‐gamma signature) were created according to the published papers.^18^, ^19^, ^20^, ^21^, ^22^ The markers of each signature are composed of several genes from the NanoString 289 panel, and the signature score for each was calculated based on the methods from those papers.

### 2.6 Differentially expressed genes and pathway analysis

Using the R package “DEseq2”, patients with primary and lymph nodes were compared for differentially expressed genes (DEGs) based on RNA‐seq data, with a threshold of log2 |fold change| >1 and False Discovery Rate (FDR) <0.05.^23^ The “pheatmap” package software was used to create DEG heatmaps. The Kyoto Encyclopedia of Genes and Genomes (KEGG) and Gene Ontology (GO) enrichment were then analyzed using the R package clusterProfiler to look into potential biological processes and signaling pathways that the significant DEGs implicated. The *p*‐values of the enrichment analysis were adjusted using the Benjamini‐Hochberg (BH) method, and a threshold of less than 0.05 was considered significant.^24^

### 2.7 Statistical analysis

The interval between the patient's verified disease relapse and the surgery was defined as the relapse‐free survival (RFS) period. The period of time between a patient's diagnosis and death was referred to as the overall survival (OS) time. Survival curves were drawn using the Kaplan–Meier method with the log‐rank *p*‐value less than 0.05 being statistically significant. The Wilcoxon test was used for TMB group difference analysis, and the Fisher's exact test was used for mutation differences between SNV and CNV groups. Fisher's exact test *p*‐values were all two‐tailed, with values less than 0.05 regarded as statistically significant. All statistical analyses were performed using the Graphpad 9.0, R 4.1 and R Bioconductor packages (https://www.r‐project.org).

## 3 RESULTS

### 3.1 Patient characteristics

**FIGURE 1:**
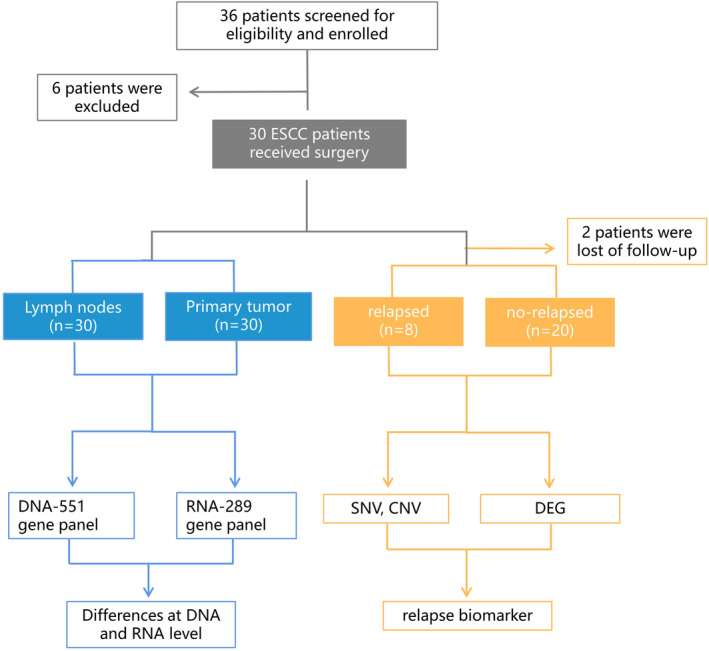
Flow chart of patient selection.

From January 2021 to February 2022, 36 patients were screened for eligibility and enrolled, and 30 patients who successfully received McKeown surgery at our hospital and had sufficient specimens were included in our study (Figure 1). A total of 60 cases were subjected to DNA‐targeted sequencing and NanoString 289‐panel RNA sequencing to identify the different molecular basis and immunological features, respectively.

### 3.2 Comparison of somatic mutation profiles in primary foci and lymph nodes

**FIGURE 2:**
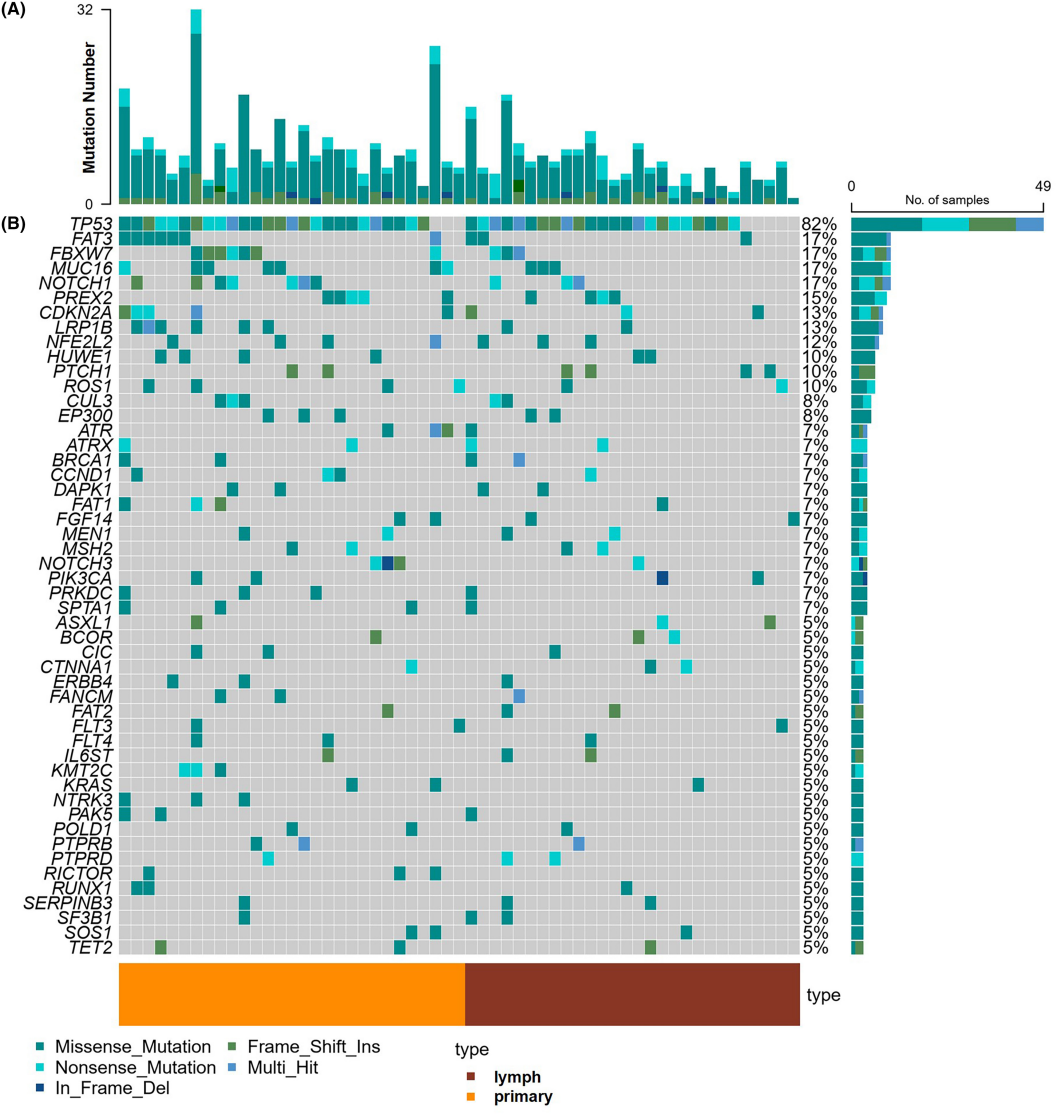
Differences of genomic mutations between primary and lymph of ESCC patients. (A) The number of genomic alterations in each patient. (B) A waterfall map of the genetic mutations in the study, light gray color in the genetic mutation waterfall diagram corresponds to the wild type or absence of any mutation, and the bottom notes the colors to represent different mutation types.

We first evaluated the association between genomic alteration using surgical primary tumor and metastatic lymph tissue. As shown in Figure 2A, the number of lymph node mutations per patient ranged from 1 to 11 (median of 4.5), and the number of mutations in the primary tumor ranged from 1 to 15 (median of 6.5). The mean number of mutations in the lymph group was lower than that in the primary group (4.5 vs. 6.4), revealed primary group had more somatic mutations. A total of 811 genomic changes (concentrated in 225 genes) were detected in the entire cohort (primary and lymph groups) by 551‐panel, and missense mutation is the most common type of mutation, followed by amplification (AMP) and nonsense mutation (Table S3). Among them, *TP53* was the most frequently altered gene, occurring in 26 (86.7%) primary tissues and 23 (76.7%) lymph nodes, followed by *FAT3* (17%), *FBXW7* (17%), *MUC16* (17%), *NOTCH1* (17%), *PREX2* (15%), *CDKN2A* (13%), and *LRP1B* (13%) (Figure 2B). There was no significant difference in somatic mutation frequency between primary tumors and lymph nodes (*p* > 0.05). In addition, RTK‐RAS, NOTCH and HRD pathways tended to be more enriched in the primary tumor group compared to lymph node group, suggesting that primary clones exhibit more susceptibility mutations (Table S4).

### 3.3 Comparison of TMB and somatic cell copy number variation in primary foci and lymph nodes

**FIGURE 3:**
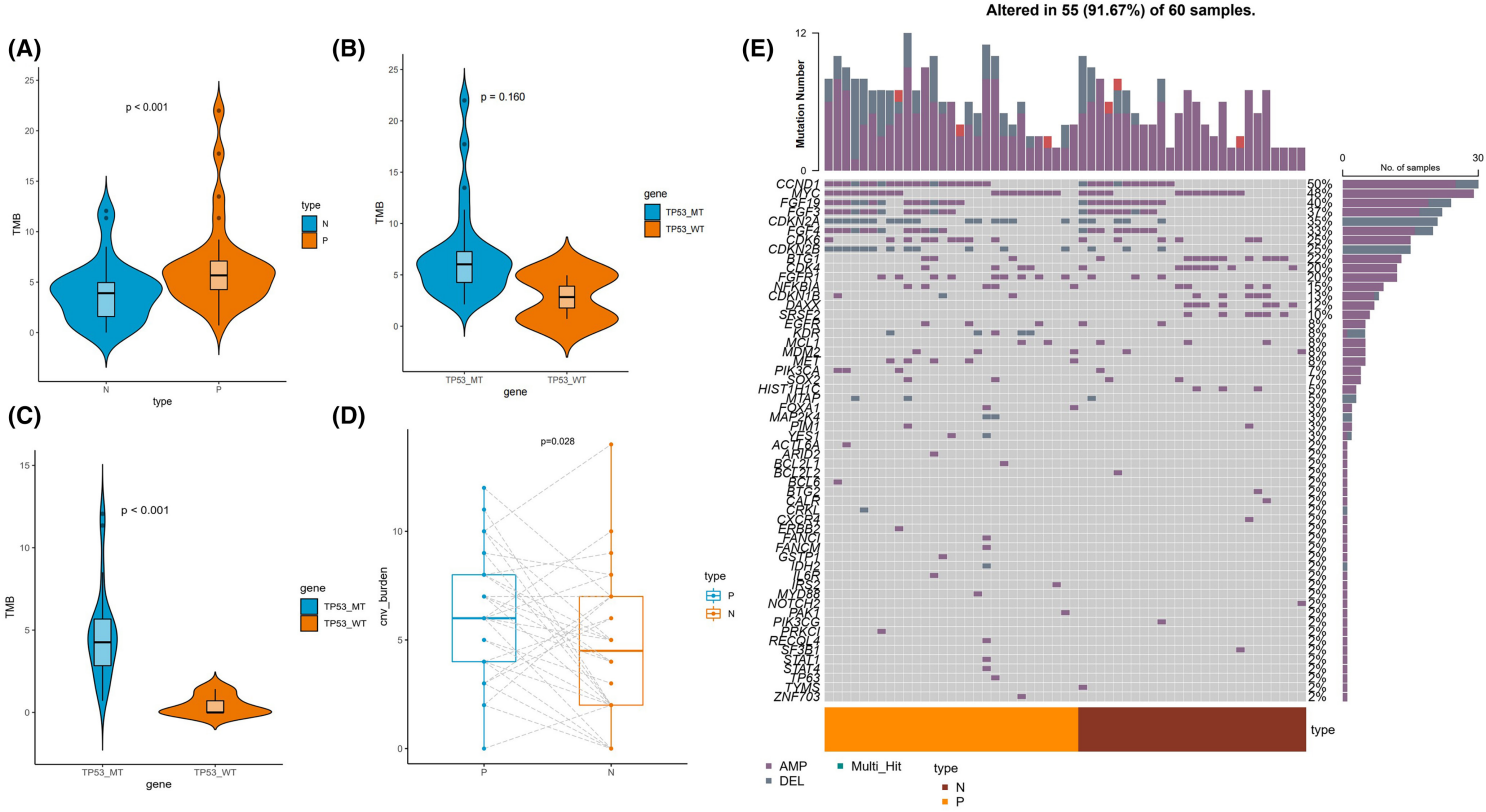
Association of tumor mutation burden (TMB) between primary tumor and lymph nodes. (A) The TMB value of primary tumor and lymph nodes group. The TMB value of *TP53*‐wild and *TP53*‐mutation group in primary tumors (B) and lymph nodes (C). (D) The CNV burden of primary tumors and lymph nodes. (E) An overview of the somatic cell copy number variation in the cohort. N, nodes; P, primary tumor.

Whether tumor mutation burden (TMB) affects the response of ESCC primary and lymph node metastases to treatment has been controversial. In this study, TMB‐H was found in 4/30 (13.3%) of the primary group and 2/30 (6.7%) of the lymph group, and all samples showed microsatellite stable (MSS) status (Table S5). The median TMB was 5.67 muts/mb in primary group and 3.91 muts/mb in lymph group (*p* < 0.001, Figure 3A). *TP53* was the most frequently mutated gene, and we further explored the relationship between *TP53* mutation and TMB of primary tumor and lymph nodes. Results revealed that there was a significant difference in TMB values between *TP53* wild and *TP53* mutant groups in lymph nodes (*p* < 0.01, Figure 3C), but no significance was observed in primary tumor (*p =* 0.16, Figure 3B).

CNV level analysis revealed that the CNV burden (sum of the number of genes in which amplification or deletion occurred) of lymph nodes was significantly lower than that of the primary tumor (*p* = 0.028, Figure 3D). As shown in Figure 3E, *CDKN2A/B* loss was significantly reduced in lymph nodes compared to primary tumor (*p* = 0.006, Table S6). *DAXX* and *SRSF2* were CNVs exclusive to lymph nodes.

### 3.4 The differences of pairwise mutual exclusivity and co‐occurrence between primary foci and lymph nodes

**FIGURE 4:**
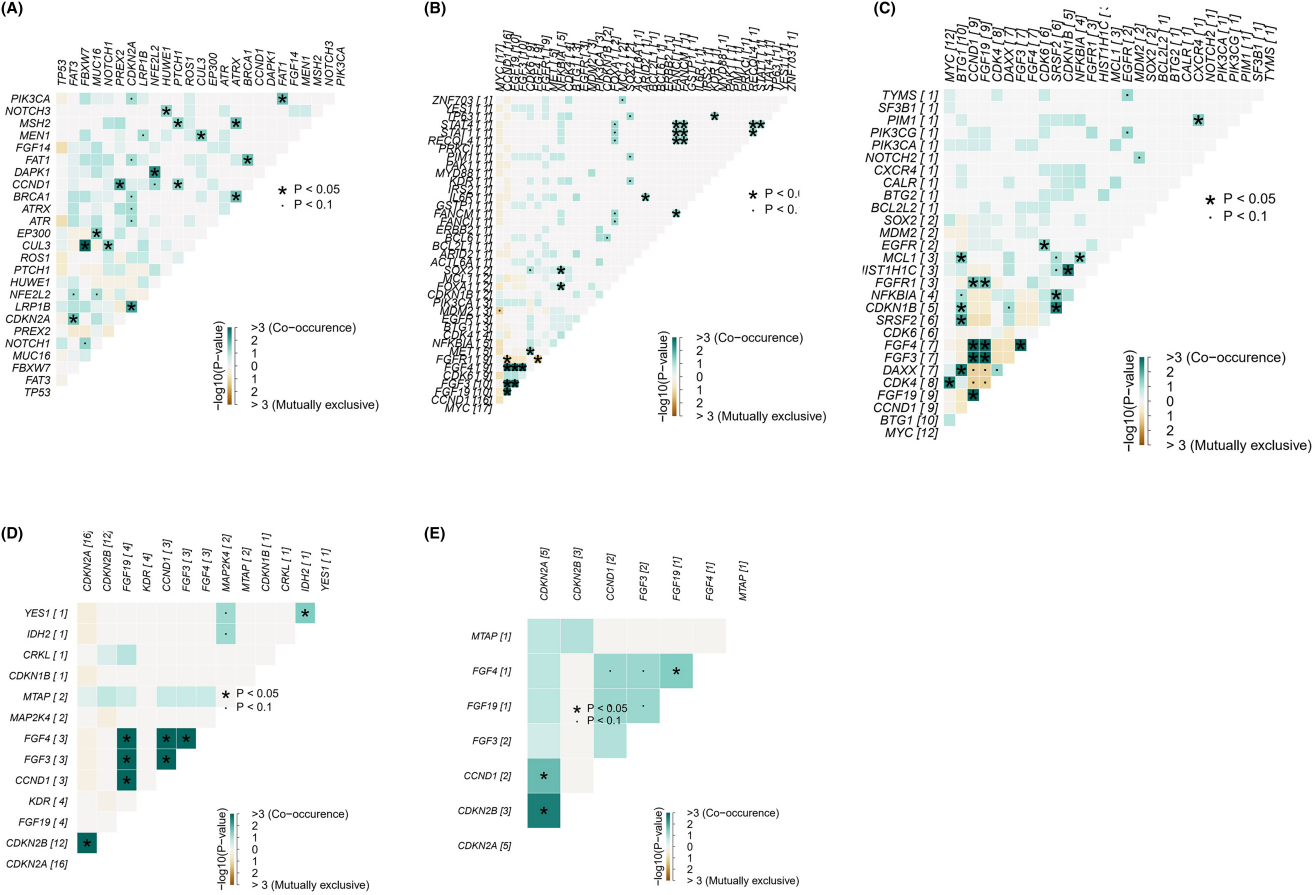
Mutation analysis of patients with ESCC. (A) Pairwise mutual co‐occurrence analysis in the cohort. Amplification co‐occurrence in primary tumor (B) and lymph nodes (C). Deletion co‐occurrence in primary tumor (D) and lymph nodes (E).

Further, we focused on genes with co‐occurrence and intergroup/relapsing differences to investigate their mechanisms of action and related pathway functions. Genes with mutation frequencies >10% are shown in Figure 4A, and *CDKN2A* deletion was co‐occurred with FAT atypical cadherin 3 (*FAT3*) (*p* < 0.05). In addition, we analyzed amplification and deletion separately in primary foci and lymph nodes. As shown in Figure 4B,C, primary foci and lymph nodes were co‐occurring in the fibroblast growth factor (FGF) family, such as FGF4 with FGF19 and FGF3 (*p* < 0.05), while lymph node specific genes *DAXX* and *SRSF2* are co‐occurred with *BTG1* (*p* < 0.05). Similarly, deletion co‐occurrence in primary foci and lymph nodes also occurs in the FGF family and CDKN2A/B, while primary foci also had YES1 and IDH2 co‐deletions not found in lymph nodes (Figure 4D,E).

### 3.5 *MUC16* mutation and NFKBIA amplification were related to decreased RFS

Magnetic resonance imaging or computed tomography were needed for clinical confirmation of the recurrence diagnosis. Considering that the median postoperative recurrence period for ESCC is 12 months,^25^, ^26^ we chose 12 months as the threshold of recurrence for ESCC. As a result, patients who experienced a local or distant recurrence within a year were categorized as belonging to the relapse group (*n* = 8), while patients who did not have a recurrence were categorized as belonging to the non‐relapse group (*n* = 20). A total of 28 patients (25 males and 3 females) with a median age of 67.5 years (44–79 years) were examined, and the subgroups age, gender, history of smoking, history of alcohol intake, and ECOG score did not significantly differ between the two groups. Table 1 displays the clinicopathological features of the patients.

**TABLE 1:** The clinicopathological features of the patients.

Characteristics	1‐year non‐relapse (*n* = 20)	1‐year relapse (*n* = 8)	*p* value*
Age (years)			
Mean (SD)	66.4 (10.1)	62.8 (8.75)	0.3627
Median (min, max)	68.0 (44.0, 79.0)	62.5 (52.0, 74.0)	
Gender, *n* (%)			
Female	1 (5.0%)	2 (25.0%)	0.3846
Male	19 (95.0%)	6 (75.0%)	
Smoking history			
Always	11 (55.0%)	2 (25.0%)	0.2337
Former	1 (5.0%)	0 (0%)	
Never	8 (40.0%)	6 (75.0%)	
Drinking history			
Always	9 (45.0%)	1 (12.5%)	0.1167
Former	2 (10.0%)	0 (0%)	
Never	9 (45.0%)	7 (87.5%)	
Eastern cooperative oncology group performance status, *n* (%)			
0	17 (85.0%)	6 (75.0%)	0.9378
1	3 (15.0%)	2 (25.0%)	
Esophageal cancer location, *n* (%)			
Down	1 (5.0%)	1 (12.5%)	1
Middle	19 (95.0%)	7 (87.5%)	
Clinical stages, *n* (%)			
IIB	1 (5.0%)	1 (12.5%)	0.2625
IIIA	3 (15.0%)	2 (25.0%)	
IIIB	16 (80.0%)	4 (50.0%)	
IVA	0 (0%)	1 (12.5%)	
T stage			
T1	1 (5.0%)	1 (12.5%)	0.7766
T2	6 (30.0%)	2 (25.0%)	
T3	13 (65.0%)	5 (62.5%)	
N stage			
N1	12 (60.0%)	4 (50.0%)	0.2713
N2	8 (40.0%)	3 (37.5%)	
N3	0 (0%)	1 (12.5%)	

* *p*‐values were calculated to compare data from indivials with and without 1‐year relapse for the folowing variables: mean ages using Student's unpaired two‐tailed *t*‐tests, others using a chi‐squared test.

**FIGURE 5:**
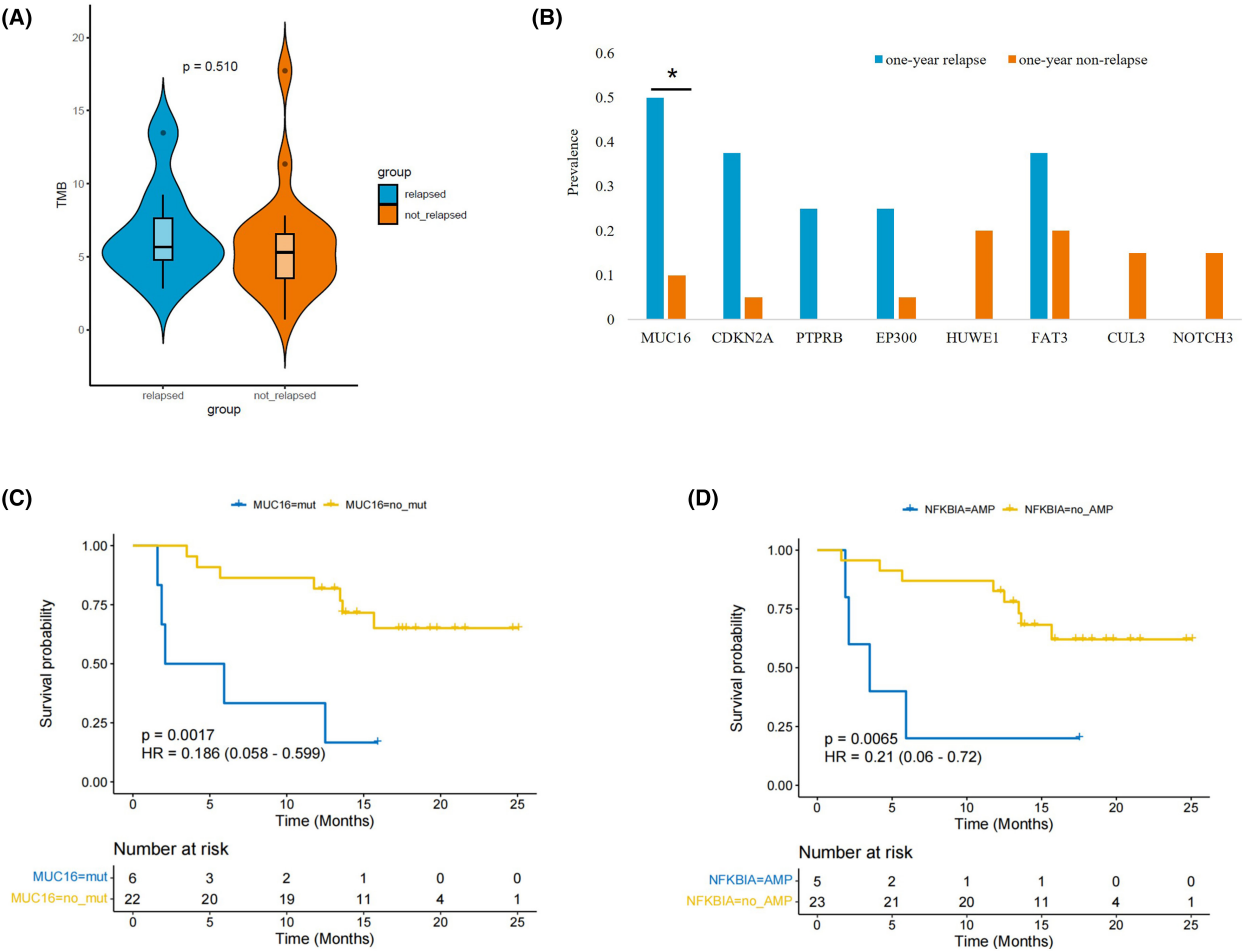
TMB and gene mutations were associated with clinical survival outcome. (A) The TMB value of relapse and not relapse in primary tumor. (B) Rate of individual somatic mutated genes between the one‐year relapse and non‐relapse groups. (C) Kaplan–Meier survival curve of *MUC16* mutation with RFS. (D) Kaplan–Meier survival curve on NFKBIA amplification with RFS.

TMB‐H has been demonstrated to be a poor predictive factor for recurrence in individuals with resected ESCC. TMB is considered connected to the quantity of neoantigens in tumors.^27^ The relationship between 1‐year relapse and TMB was analyzed, and no significant difference in TMB values was observed between the relapse and non‐relapse groups in primary tumor (Figure 5A). The primary tumor somatic *MUC16* mutation rate was statistically different between the one‐year recurrent and non‐recurrent groups (Figure 5B, 50% vs. 5.3%, *p* = 0.038, OR = 8.1). Notably, Kaplan–Meier survival curve indicated that *MUC16* mutation presented significantly shorter RFS (Figure 5C, HR = 0.186, 95% CI 0.058–0.599, *p* = 0.0017).

We also explored factors associated with recurrence between the two groups at the CNV level. Results showed that NFKBIA (*p* = 0.015, OR = 16.36) and FGF19 (*p* = 0.038, OR = 0.10) mutations of primary tumor were significantly different between the two groups, while only FGF19 (*p* = 0.0292) in lymph nodes. When the two mutation types of deletion (DEL) or AMP were counted separately, the gene NFKBIA was the only one found to be significantly amplified in the primary tumor showing significant mutation difference between the relapse and not‐relapse groups. Patients with NFKBIA amplification had significantly shorter RFS, as shown by the Kaplan–Meier survival curve (Figure 5D, *p* = 0.0056, HR = 0.21, 95% CI: 0.06–0.72).

### 3.6 The *MUC16* mutation's independent prognostic significance in disease relapse

Prognostic factors may include clinical characteristics and genetic changes. We investigated the relationship between patients' RFS and genetic alterations using a univariate Cox regression model. Age, sex, history of drinking and smoking, and clinical stage were among the baseline clinical factors that were examined. Univariate analysis results indicated that *MUC16* mutation (HR: 5.39, 95% CI: 1.67–17.4, *p* = 0.005) was an adverse prognostic factor for RFS (Table 2). These results suggest that *MUC16* mutation in the primary tumor is a poor prognostic factor for ESCC patients with LNM, and those patients with LNM harboring *MUC16* mutation have a higher risk of relapse. Variables that had a *p*‐value of less than <0.2 in the univariate analysis were included in the multivariate analysis to see if the *MUC16* mutation was an independent predictor of survival outcomes. Multivariate analysis results revealed that the *MUC16* mutation was an independent genetic factor substantially linked with shorter RFS (HR: 7.36, 95% CI: 1.79–30.23, *p* = 0.006).

**TABLE 2:** The results of univariate analysis on *MUC16* mutation with RFS.

	Univarible			Mulivariable		
Characteristic	HR	95% CI	*p* value	HR	95% CI	*p* value
Age group (years)						
<65						
≥65	0.9	0.27–2.99	0.86			
Sex_group						
Female						
Male	0.31	0.08–1.17	0.09	4.92	0.91–26.54	0.064
Smoking history						
Never						
Always	0.29	0.08–1.07	0.06	1.62	0.37–7.09	0.522
Drinking history						
Never						
Always	0.48	0.13–1.81	0.28			
T stage						
T2						
T3	1.11	0.43–2.85	0.83			
N stage						
N2						
N3	1.30	0.50–3.37	0.59			
*MUC16*						
Wild type						
Mutation type	5.39	1.67–17.4	0.005	7.36	1.79–30.23	0.006

Abbreviations: CI, confidence interval; HR, hazard ratio.

### 3.7 Analysis of immune cell profiles and identification of genes with differential expression in relapse and non‐relapse groups

289‐panel transcriptome analysis of 29 primary tumor samples and 30 lymph nodes was performed, and many differences in immune‐related genes, immune cell abundance, and immune scores were found between primary foci and lymph nodes (Figure S1), suggesting lymph nodes had better immune micro‐infiltration than primary foci.

**FIGURE 6:**
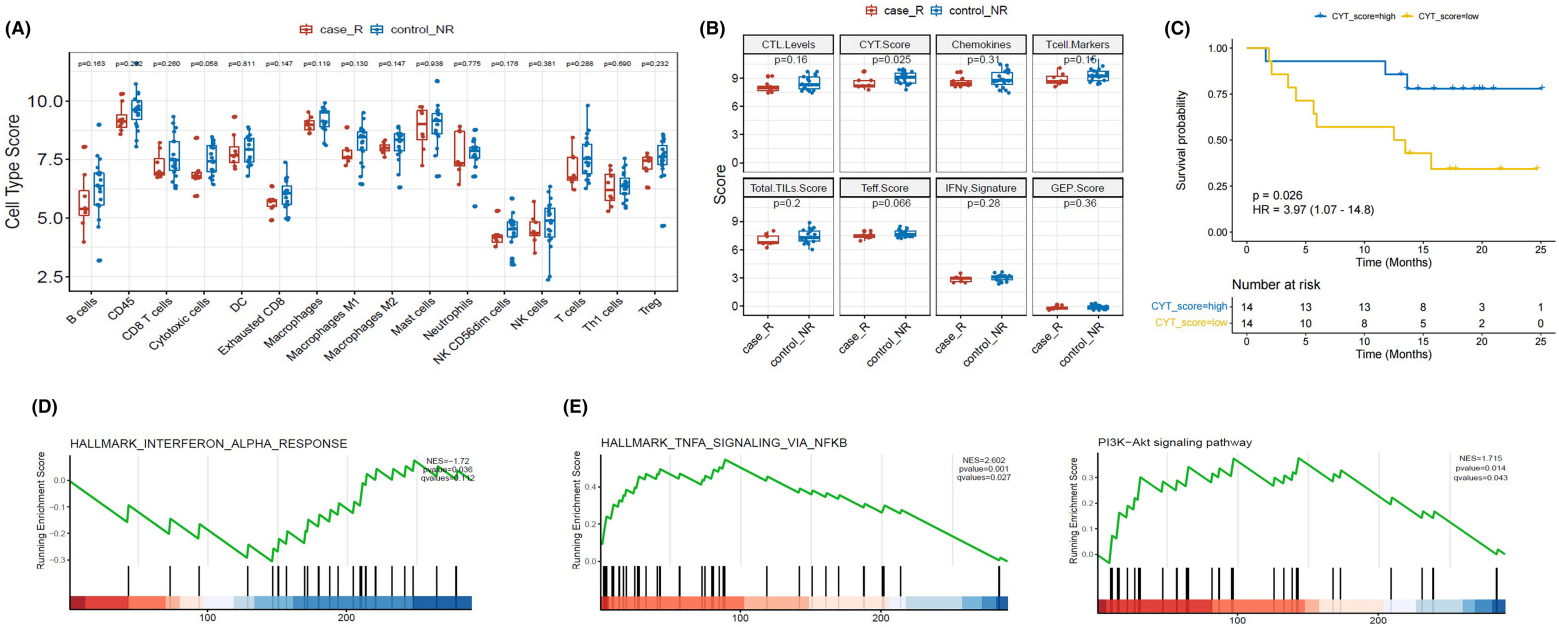
Identification of DEGs and immune cell profile analyses in relapse and non‐relapse groups. (A) The immune cell composition assessment shown in box‐plot of immune cell in relapse group (*n* = 8) and non‐relapse group (*n* = 19). (B) The statistical analysis of seven gene sets in relapse group and non‐relapse groups. (C) Kaplan–Meier survival curve of CYT score with RFS. (D) IFN‐α pathway was significantly enriched in the relapse group analyzed by GSEA. (E) TNF‐α/NF‐κB and PI3K‐Akt pathway was significantly enriched in the non‐relapse group analyzed by GSEA.

We also examined the fluctuation of TiME anti‐tumor immunity in baseline tumor tissues of recurrence and non‐recurrence groups in order to clarify the role of TiME in postoperative relapse of ESCC. We first discussed the differences in immune cells between the two groups. As shown in Figure 6A, a cell type with an up‐regulation trend was identified in the non‐relapse group, namely cytotoxic cells, which is associated with tumor cell killing. CYT score was also higher than relapse group (*p* = 0.025), further demonstrating a better infiltration of the inflammatory tumor microenvironment in the non‐relapse group (Figure 6B). Further, we grouped according to the median CYT score and found that higher CYT score had significantly longer RFS (Figure 6C, *p* = 0.026, HR = 3.97, 95% CI: 1.07–14.8). In addition, we analyzed the function of DEGs through GSEA enrichment, and it was found that DEGs in the relapse group are enriched in the TNF‐α/NF‐κB and inflammatory response pathways (Figure 6D), and DEGs in the non‐relapse group are concentrated in the interferon α (IFN‐α) response pathways (Figure 6E).

## 4 DISCUSSION

Although the primary and lymph node responses to treatment of ESCC are inconsistent, and patients with LNM have a poor prognosis, the genetic and immunological characteristics of relapse in this subset of patients have not been well described.^28^ Our study compared the molecular characteristics of the primary tumors and lymph nodes, and was the first to identify the molecular basis and immunological characteristics of relapse in lymph node‐positive ESCC. The results showed that *MUC16* mutation and a lower immune response were associated with reduced RFS in lymph node‐positive ESCC.

We performed next generation sequencing of paired tumor and lymph node tissues from 30 ESCC patients in this study. Previous studies have found that *TP53*, *PIK3CA*, *NOTCH1*, *FAT1*, *CDKN2A*, and *EP300* are the top mutations in Chinese ESSC population.^29^, ^30^ Consistent with previous reports, *TP53* was the top‐ranked mutation in our cohort, with mutation frequencies of 86.7% and 76.7% in the primary tumor and lymph nodes, respectively. There was no significant difference in gene mutation frequency between primary tumors and lymph nodes, while the RTK‐RAS, NOTCH and HRD pathways tended to be more enriched in the primary tumor group compared to lymph node group. This suggests that primary clones exhibited more sensitive mutations.

Elevated TMB is accompanied by an increase in the number and frequency of neoantigen production and the immunogenicity of the organism, and TMB has been reported as a potential predictor for tumor behaviors. TMB varies widely between different cancer classes, ranging from 0.001 muts/mb to more than 400 muts/mb, and the average TMB is at 6.7 muts/mb for esophageal adenocarcinoma (EAC) and 6.4 muts/mb for ESCC.^31^ In our study, the median TMB was 5.67 muts/mb in primary tumor group and 3.91 muts/mb in lymph nodes group. Lymph node TMB was generally lower than that of the primary tumor, suggesting that the efficacy of ESCC lymph nodes receiving immunotherapy may be inferior to that of the primary lesion, which is consistent with the reports of breast cancer and lung cancer.^32^, ^33^

A growing body of research indicates that the tumor microenvironment is essential for immune response regulation, immune escape promotion, angiogenesis facilitation, and metastasis induction.^34^, ^35^ Numerous measures have been found to describe the ability of tumor cells to trigger adaptive immunological responses, the immune activation's downstream effects, like the existence of immune cells, and the immune scores' gene expression profile. In this study, there were significant differences in the abundance of immune cells and immunoinfiltration scores between the primary lesion and lymph nodes, similar to the huge differences in TMB values between the two, which may be caused by the characteristics of the tissue itself.

In our study, higher CYT score was associated with a favorable prognosis of node‐positive ESCC. The immunocytolytic score (CYT score) is the cytolytic activity of immune infiltration, defined as the logarithmic average (geometric mean) of the perforation protein (PRF1) and granzyme A (GZMA) expression values, provided by Rooney et al.^36^ Moreover, high expression of CYT markers is associated with improved prognosis of pan‐cancer, which is consistent with our findings. We also compared DEGs enrichment in the relapsed and non‐relapsed groups, and DEGs enrichment in the IFN‐γ pathway was observed in patients with a good prognosis in our cohort. IFN‐γ is produced by immune cells and is used in anti‐tumor therapy due to its cellular differentiation and growth‐regulating properties. Previous studies have reported that an enriched IFN‐γ pathway predicts better prognosis in patients with colorectal cancer and breast cancer, which is consistent with our results.^37^, ^38^

Through univariate and multivariate analysis, it was found that *MUC16* mutation was an independent predictor of one‐year recurrence for ESCC. The *MUC16* protein is a highly glycosylated transmembrane protein whose coding gene is located in the 19p13.2 region and secretes the extracellular portion through hydrolysis of a specialized site in the near‐membrane portion (CA125).^39^ *MUC16* is often mutated in ovarian cancer, gastric cancer and other tumor tissues.^40^, ^41^ In ESCC, the mutation frequency of the *MUC16* gene was about 14%, similar to 17.7% in our study. In addition, there is a high expression of CA125 protein encoded by *MUC16* is associated with metastasis and poor prognosis of epithelial ovarian cancer.^41^ Higashi et al. found that *MUC16* was not expressed in normal liver tissues and intrahepatic bile duct tissues, but was highly expressed in extrahepatic cholangiocarcinoma cells, suggesting a poor prognosis.^42^ In contrast to ovarian and cholangiocarcinoma, Li et al. found that *MUC16* mutation may be associated with higher tumor mutation load and better survival outcomes in gastric cancer, possibly because the mutation is not necessarily lead to overexpression.^40^ However mutation in *MUC16* in tissues and the prognosis of ESCC have not yet been clarified. In our cohort, we found *MUC16* mutation in primary tumor associated with decreased RFS. As follow‐up is not yet mature, we further verified this using the ESCC cohort in the TCGA database (*n* = 96), and found *MUC16* mutation has a tendency to shorten OS (*p* = 0.094, Figure S2), which is consistent with our finding. These results strongly suggest that *MUC16* mutation is associated with poorer survival outcomes in ESCC.

There are some limitations in this study. First, findings may be influenced by other factors, such as inter‐tumor and intra‐tumor heterogeneity, and differences in detection of different batches of samples. Second, there are limitations in the detection and analysis techniques and the sample size of this study is small. In the future, more advanced assays with larger sample sizes are needed to validate these findings and explore other potential factors that could influence the results.

## 5 CONCLUSION

Our findings provide evidence that differences in the molecular and immune characteristics of ESCC primary tumors and lymph nodes. This study also reveals that *MUC16* mutation and lower CYT score are associated with decreased RFS in patients who were node‐positive ESCC patients, providing important clues for personalized treatment and monitoring, and offering new ideas for improving the prognosis of these patients.

## AUTHOR CONTRIBUTIONS

**Hua‐guang Pan:** Conceptualization (equal); methodology (equal); supervision (equal); validation (equal). **Han‐lin Fang:** Investigation (equal); validation (equal). **Chan Zhu:** Data curation (equal); project administration (equal); writing – original draft (equal). **Si Li:** Methodology (equal); validation (equal). **Huan Yi:** Formal analysis (equal); visualization (equal). **Xing Zhang:** Formal analysis (equal); supervision (equal). **Xiang‐yu Yin:** Formal analysis (equal); visualization (equal). **Yun‐jie Song:** Formal analysis (equal). **Dongsheng Chen:** Validation (equal). **Chun‐tong Yin:** Data curation (equal); project administration (equal); writing – original draft (equal).

## CONFLICT OF INTEREST STATEMENT

C. Zhu, S. Li, H. Yi, X. Zhang, X. Yin, Y. Song and D. Chen are employed by Jiangsu Simcere Diagnostics Co., LTD.

## ETHICS STATEMENT

This study has been approved by the Ethics Committee of the First Affiliated Hospital of Anhui Medical University (PJ2022‐11‐13) in compliance with the ethical principles stated by the Declaration of Helsinki.

## CONSENT

Patient consent was waived due to the reason of retrospective design without changing the patients' therapy.

## Supporting information


Data S1.

## Data Availability

All data supporting the findings of this study are available within the paper and its supplementary information.
