# Supplementary material for: Molecular and immunological characteristics of postoperative relapse in lymph node‐positive esophageal squamous cell cancer
Source: Cancer Med. 2024 May 11;13(9):e7228. doi: 10.1002/cam4.7228 (PMC11087845; doi:10.1002/cam4.7228)

**Supplementary Materials**

**Supplementary Table 1: TME 289 gene list**

**Supplementary Table 2: Cell type gene list**

**Supplementary Table 3: Overview of gene mutation summary**

**Supplementary Table 4: Fisher test analysis of pathway differences (without CNV)**

**Supplementary Table 5: Overview of TMB and MSI**

**Supplementary Table 6: Fisher test analysis of differences in mutations in CNV**

**Supplementary Table 7: Patient baseline characteristics**

**Supplementary Figure 1: Identification of DEGs and immune cell profile analyses in primary tumor and lymph nodes.** (A) Transcriptome analysis on differential expression between primary tumor (n=29) and lymph nodes (n=30). (B) Enrichment pathways of differentially expressed genes. The immune cell composition assessment shown in heatmap (C) and the box-plot of immune cell (D). (E) The statistical analysis of seven gene sets in primary tumor and lymph node groups.

**Supplementary Figure 2: K-M survival curve of *MUC16* mutation with OS in TCGA database (n=96).**

**Supplementary Table 1: TME 289 gene list**

| ABCF1 | CD44 | FCGR1A | IL21R | NFKBIA | TIE1 |
| --- | --- | --- | --- | --- | --- |
| ADM | CD47 | FCGR2B | IL2RA | NKG7 | TIGIT |
| ADORA2A | CD48 | FCRL2 | IL2RB | NOS2 | TLR3 |
| AKT1 | CD6 | FGF13 | IL2RG | NT5E | TLR7 |
| ANGPT2 | CD68 | FOXP3 | IL4 | OAS1 | TLR8 |
| ARG1 | CD69 | FPR1 | IL6 | OAS2 | TLR9 |
| ATM | CD70 | FUT4 | IL7R | OAS3 | TNF |
| AXL | CD74 | G6PD | IRF1 | PDCD1 | TNFRSF14 |
| BCL2 | CD79A | GBP1 | IRF4 | PDCD1LG2 | TNFRSF17 |
| BIRC5 | CD79B | GNLY | IRF9 | PDGFA | TNFRSF18 |
| BLK | CD80 | GUSB | ISG15 | PDGFB | TNFRSF1A |
| BLM | CD84 | GZMA | ITGA1 | PECAM1 | TNFRSF1B |
| BRCA1 | CD86 | GZMB | ITGAE | PIK3CA | TNFRSF4 |
| BRCA2 | CD8A | GZMH | ITGAL | PIK3CD | TNFRSF9 |
| BRIP1 | CD8B | GZMK | ITGAM | PMS2 | TNFSF10 |
| BTLA | CDKN2A | HAVCR2 | ITGAX | PNOC | TNFSF13B |
| C1QA | CEACAM3 | HDC | ITGB2 | POLR2A | TNFSF18 |
| C1QB | CMKLR1 | HERC6 | KIR2DL3 | PRF1 | TNFSF4 |
| CCL13 | CPA3 | HIF1A | KIR3DL1 | PSMB10 | TNFSF9 |
| CCL18 | CSF1R | HLA-DMA | KIR3DL2 | PSMB9 | TRAT1 |
| CCL2 | CSF2 | HLA-DMB | KLRB1 | PTEN | TWIST1 |
| CCL20 | CSF2RB | HLA-DOA | KLRD1 | PTGER4 | VCAM1 |
| CCL21 | CSF3R | HLA-DOB | KLRK1 | PTGS2 | VEGFA |
| CCL22 | CTAG1B | HLA-DPA1 | LAG3 | PTPN11 | VTCN1 |
| CCL4 | CTLA4 | HLA-DQA2 | LCK | PTPRC | ZAP70 |
| CCL5 | CTSS | HLA-DRA | LILRB2 | PVR | ZEB1 |
| CCL7 | CTSW | HSD11B1 | LY9 | RAD51 | CXCL2 |
| CCND1 | CX3CL1 | ICAM1 | LYZ | RB1 | FCGR3B |
| CCR2 | CX3CR1 | ICOS | MAGEA1 | RORC | GZMM |
| CCR4 | CXCL1 | ICOSLG | MAGEA12 | RUNX3 | HLA-DQA1 |
| CCR5 | CXCL10 | IDO1 | MAGEA4 | S100A12 | HLA-DRB1 |
| CD14 | CXCL11 | IFI27 | MAGEC2 | S100A8 | HLA-E |
| CD163 | CXCL12 | IFI35 | MELK | S100A9 | OAZ1 |
| CD19 | CXCL13 | IFI6 | MKI67 | SDHA | PF4 |
| CD1C | CXCL5 | IFIH1 | MLANA | SELL | PRR5 |
| CD2 | CXCL8 | IFIT1 | MLH1 | SH2D1A | STK11IP |
| CD209 | CXCL9 | IFIT2 | MMP9 | SIGLEC5 | TBC1D10B |
| CD244 | CXCR2 | IFIT3 | MRC1 | SLAMF7 | TPSAB1 |
| CD247 | CXCR3 | IFITM1 | MS4A1 | SNAI1 | UBB |
| CD27 | CXCR4 | IFITM2 | MS4A2 | SPIB |  |
| CD274 | CXCR6 | IFNG | MS4A4A | STAT1 |  |
| CD276 | CYBB | IL10 | MSH2 | STAT3 |  |
| CD28 | DLL4 | IL10RA | MSH6 | STAT4 |  |
| CD38 | EGFR | IL12RB2 | MTOR | TAP1 |  |
| CD3D | EIF2AK2 | IL15 | MX1 | TBP |  |
| CD3E | ENTPD1 | IL17A | MYC | TBX21 |  |
| CD3G | EOMES | IL18 | NBN | TCL1A |  |
| CD4 | FAS | IL1A | NCAM1 | TDO2 |  |
| CD40 | FASLG | IL1B | NCR1 | TFRC |  |
| CD40LG | FCAR | IL2 | NECTIN2 | TGFB1 |  |

**Supplementary Table 2: Cell type gene list**

| **Cell type** | **Gene list** |
| --- | --- |
| T cells | CD3D、CD3E、CD3G、CD6、SH2D1A、TRAT1 |
| B cells | BLK、CD19、FCRL2、MS4A1、PNOC、SPIB、TCL1A、TNFRSF17 |
| Mast cells | CPA3、HDC、MS4A2 |
| DC | CCL13、CD209、HSD11B1 |
| Macrophages | CD163、CD68、CD84、MS4A4A |
| Neutrophils | CEACAM3、CSF3R、FCAR、FPR1、S100A12、SIGLEC5 |
| Cytotoxic cells | CTSW、GNLY、GZMA/B/H、KLRB1、KLRD1、KLRK1、NKG7、PRF1 |
| Exhausted CD8 | PTGER4、LAG3、EOMES、CD244 |
| NK CD56 cell | KIR3DL1/2/3、IL21R |
| CD8 T cell | CD8B、CD8A |
| CD45 cell | PTPRC |
| Th1 cell | TBX21 |
| NK cell | NCR1 |
| Treg cell | FOXP3 |

**Supplementary Table 3: Overview of gene mutation summary**

| ID | summary | Mean | Median |
| --- | --- | --- | --- |
| NCBI_Build |  |  |  |
| Center |  |  |  |
| Samples | 60 |  |  |
| nGenes | 225 |  |  |
| AMP | 251 | 4.183 | 4 |
| DEL | 68 | 1.133 | 0 |
| Frame_Shift_Ins | 47 | 0.783 | 1 |
| In_Frame_Del | 8 | 0.133 | 0 |
| In_Frame_Ins | 3 | 0.05 | 0 |
| Missense_Mutation | 361 | 6.017 | 5 |
| Nonsense_Mutation | 73 | 1.217 | 1 |
| total | 811 | 13.517 | 13.5 |

**Supplementary Table 4: Fisher test analysis of pathway differences (without CNV)**

| Hugo_Symbol | N | P | pval | or | ci.up | ci.low | adjPval |
| --- | --- | --- | --- | --- | --- | --- | --- |
| RTK-RAS | 11 | 23 | 0.003756338 | 0.182007724 | 0.614403258 | 0.048490756 | 0.037563376 |
| NOTCH | 9 | 17 | 0.067278508 | 0.334159776 | 1.06897381 | 0.098312929 | 0.336392541 |
| HRD | 6 | 13 | 0.09461078 | 0.333247831 | 1.170299349 | 0.085436377 | 0.09461078 |
| DDR | 24 | 29 | 0.102789717 | 0.141962796 | 1.294273446 | 0.002906193 | 0.102789717 |
| Cell_Cycle | 24 | 28 | 0.254214336 | 0.291465053 | 1.827759446 | 0.026422319 | 0.569298538 |
| TP53 | 23 | 27 | 0.298979382 | 0.371150751 | 1.859758817 | 0.05555436 | 0.569298538 |
| MYC | 12 | 17 | 0.301467123 | 0.515666437 | 1.599359938 | 0.16100049 | 0.569298538 |
| PI3K | 6 | 10 | 0.381654119 | 0.50583927 | 1.857982279 | 0.127009795 | 0.569298538 |
| Hippo | 7 | 11 | 0.398508977 | 0.531396424 | 1.850168419 | 0.143718256 | 0.569298538 |
| NRF2 | 6 | 8 | 0.761068497 | 0.691818268 | 2.69529302 | 0.168459472 | 0.951335621 |
| WNT | 1 | 2 | 1 | 0.488464946 | 9.877566157 | 0.00792058 | 1 |
| TGF-Beta | 2 | 2 | 1 | 1 | 14.69198308 | 0.068064331 | 1 |

**Supplementary Table 5: Overview of TMB and MSI**

| **order_number** | **sample_number** | **TMB** | **MSI_status** | **group** | **order_number** | **sample_number** | **TMB** | **MSI_status** | **group** |
| --- | --- | --- | --- | --- | --- | --- | --- | --- | --- |
| 220407226 | TIS74110489F1D1L1 | 4.26 | MSS | P | 220407303 | TIS69110488F1D1L1 | 4.96 | MSS | L |
| 220407311 | TIS44110490F1D2L1 | 3.55 | MSS | P | 220407308 | TIS75110486F1D2L1 | 4.96 | MSS | L |
| 220407316 | TIS62123974F1D2L1 | 2.84 | MSS | P | 220409207 | TIS41110482F1D2L1 | 3.55 | MSS | L |
| 220409764 | TIS11110485F1D1L1 | 7.09 | MSS | P | 220411132 | TIS65124068F1D1L1 | 3.55 | MSS | L |
| 220411154 | TIS89123977F1D1L1 | 6.38 | MSS | P | 220411157 | TIS25123976F1D2L1 | 4.26 | MSS | L |
| 220411160 | TIS25123979F1D1L1 | 7.8 | MSS | P | 220411161 | TIS52123980F1D2L1 | 1.42 | MSS | L |
| 220411693 | TIS75123982F1D1L1 | **11.35** | MSS | P | 220411692 | TIS72124070F1D2L1 | **12.06** | MSS | L |
| 220411709 | TIS12123984F1D2L1 | 9.22 | MSS | P | 220411708 | TIS29123986F1D1L1 | 5.67 | MSS | L |
| 220411717 | TIS91123988F1D1L1 | 0.71 | MSS | P | 220411719 | TIS46123989F1D2L1 | 4.96 | MSS | L |
| 220411735 | TIS10123993F1D2L1 | 5.67 | MSS | P | 220411737 | TIS22123992F1D1L1 | 4.26 | MSS | L |
| 220411753 | TIS74123996F1D1L1 | 2.84 | MSS | P | 220411754 | TIS81124071F1D2L1 | 2.13 | MSS | L |
| 220411825 | TIS43123997F1D1L1 | 2.13 | MSS | P | 220411826 | TIS54125674F1D1L1 | 2.13 | MSS | L |
| 220412059 | TIS15124004F1D1L1 | 4.26 | MSS | P | 220412064 | TIS24124043F1D2L1 | 0.71 | MSS | L |
| 220412105 | TIS31124007F1D2L1 | **21.99** | MSS | P | 220412108 | TIS34124008F1D1L1 | 1.42 | MSS | L |
| 220412115 | TIS96124010F1D1L1 | 7.09 | MSS | P | 220412123 | TIS85124011F1D2L1 | 6.38 | MSS | L |
| 220412141 | TIS58124013F1D1L1 | 6.38 | MSS | P | 220412157 | TIS81125665F1D1L1 | 4.96 | MSS | L |
| 220412156 | TIS13124016F1D1L1 | **17.73** | MSS | P | 220412173 | TIS93125745F1D1L1 | 0.71 | MSS | L |
| 220412172 | TIS93124019F1D1L1 | 4.96 | MSS | P | 220412180 | TIS74124023F1D2L1 | 2.84 | MSS | L |
| 220412179 | TIS79124022F1D1L1 | 5.67 | MSS | P | 220412496 | TIS82124014F1D2L1 | 5.67 | MSS | L |
| 220412791 | TIS68124027F1D1L1 | 4.26 | MSS | P | 220412792 | TIS10124028F1D2L1 | 2.84 | MSS | L |
| 220412802 | TIS83124036F1D1L1 | **13.48** | MSS | P | 220412803 | TIS17124037F1D2L1 | **11.35** | MSS | L |
| 220412808 | TIS83124033F1D1L1 | 6.38 | MSS | P | 220412812 | TIS33124034F1D2L1 | 3.55 | MSS | L |
| 220412818 | TIS43124030F1D1L1 | 4.26 | MSS | P | 220412819 | TIS99124031F1D2L1 | 4.26 | MSS | L |
| 220506254 | TIS63125767F1D1L1 | 6.38 | MSS | P | 220506255 | TIS20125615F1D1L1 | 4.26 | MSS | L |
| 220506259 | TIS36125765F1D1L1 | 3.55 | MSS | P | 220506260 | TIS61125763F1D1L1 | 1.42 | MSS | L |
| 220506264 | TIS12125611F1D1L1 | 4.96 | MSS | P | 220506263 | TIS84125607F1D1L1 | 0.0 | MSS | L |
| 220506267 | TIS69125659F1D1L1 | 7.8 | MSS | P | 220506268 | TIS45125658F1D1L1 | 8.51 | MSS | L |
| 220506271 | TIS89125760F1D1L1 | 6.38 | MSS | P | 220506274 | TIS47125762F1D1L1 | 5.67 | MSS | L |
| 220506275 | TIS10125621F1D1L1 | 2.84 | MSS | P | 220506276 | TIS45125623F1D1L1 | 0.0 | MSS | L |
| 220604188 | TIS15125601F1D1L1 | 5.67 | MSS | P | 220604170 | TIS91125755F1D1L1 | 0.0 | MSS | L |

**Supplementary Table 6: Fisher test analysis of differences in mutations in CNV**

| Hugo_Symbol | N | P | pval | or | ci.up | ci.low | adjPval |
| --- | --- | --- | --- | --- | --- | --- | --- |
| CDKN2A | 5 | 16 | 0.006107225 | 0.180592811 | 0.655516826 | 0.042196381 | 0.137984595 |
| DAXX | 7 | 0 | 0.010542535 | #NUM! | #NUM! | 1.649704748 | 0.137984595 |
| CDKN2B | 3 | 12 | 0.015331622 | 0.171776749 | 0.758606043 | 0.027276659 | 0.137984595 |
| SRSF2 | 6 | 0 | 0.023720704 | #NUM! | #NUM! | 1.30287157 | 0.160114751 |
| KDR | 0 | 5 | 0.052185549 | 0 | 1.01719355 | 0 | 0.221655626 |
| MET | 0 | 5 | 0.052185549 | 0 | 1.01719355 | 0 | 0.221655626 |
| BTG1 | 10 | 3 | 0.057466273 | 4.38966116 | 28.01792075 | 0.963489916 | 0.221655626 |
| CCND1 | 11 | 19 | 0.06984871 | 0.341658114 | 1.077532718 | 0.102626975 | 0.235739396 |
| FGFR1 | 3 | 9 | 0.104164737 | 0.265066619 | 1.236027294 | 0.041044621 | 0.31249421 |
| HIST1H1C | 3 | 0 | 0.237288136 | #NUM! | #NUM! | 0.421860231 | 0.640677966 |
| MYC | 12 | 17 | 0.301467123 | 0.515666437 | 1.599359938 | 0.16100049 | 0.737288136 |
| CDK4 | 8 | 4 | 0.333411738 | 2.330162074 | 12.05520374 | 0.535538758 | 0.737288136 |
| FGF4 | 8 | 12 | 0.411756597 | 0.551038883 | 1.842583079 | 0.157277577 | 0.737288136 |
| FGF3 | 9 | 13 | 0.421975381 | 0.565923334 | 1.8358714 | 0.167985802 | 0.737288136 |
| FGF19 | 10 | 14 | 0.429569193 | 0.576833763 | 1.830632258 | 0.17610078 | 0.737288136 |
| FOXA1 | 0 | 2 | 0.491525424 | 0 | 5.2956279 | 0 | 0.737288136 |
| MAP2K4 | 0 | 2 | 0.491525424 | 0 | 5.2956279 | 0 | 0.737288136 |
| YES1 | 0 | 2 | 0.491525424 | 0 | 5.2956279 | 0 | 0.737288136 |
| CDK6 | 6 | 9 | 0.552003431 | 0.588622159 | 2.217705675 | 0.14586759 | 0.784425929 |
| PIK3CA | 1 | 3 | 0.611953613 | 0.316016201 | 4.211021402 | 0.005723854 | 0.826137377 |
| CDKN1B | 5 | 3 | 0.706469199 | 1.782600382 | 12.68194494 | 0.309391045 | 0.908317542 |
| MTAP | 1 | 2 | 1 | 0.488464946 | 9.877566157 | 0.00792058 | 1 |
| EGFR | 2 | 3 | 1 | 0.647569237 | 6.120008415 | 0.050403133 | 1 |
| MCL1 | 3 | 2 | 1 | 1.54423642 | 19.84003648 | 0.163398468 | 1 |
| MDM2 | 2 | 3 | 1 | 0.647569237 | 6.120008415 | 0.050403133 | 1 |
| NFKBIA | 4 | 5 | 1 | 0.772600498 | 4.055124736 | 0.136632889 | 1 |
| SOX2 | 2 | 2 | 1 | 1 | 14.69198308 | 0.068064331 | 1 |

**Supplementary Table 7: Patient baseline characteristics**

| **ID** | **Age** | **Sex** | **Smoking history** | **Drinking history** | **ECOG** | **Location** | **cTNM** | **I-IV Stages** | **Differentiation** | **Resection** | **pTNM** |
| --- | --- | --- | --- | --- | --- | --- | --- | --- | --- | --- | --- |
| 1 | 68 | Male | Always | Always | 0 | Middle | cT3N0M0 | IVA | G2 | R0 | pT3N3M0 |
| 2 | 79 | Male | Never | Always | 0 | Down | cT3N2M0 | ⅢB | G2 | R0 | pT3N2M0 |
| 3 | 70 | Male | Always | Never | 0 | Middle | cT2N0M0 | ⅢA | G2 | R0 | pT2N1M0 |
| 4 | 68 | Female | Never | Never | 0 | Middle | cT2N0M0 | ⅢB | G2 | R0 | pT2N2M0 |
| 5 | 67 | Male | Never | Never | 0 | Middle | cT3N0M0 | ⅢB | G2 | R0 | pT3N2M0 |
| 6 | 58 | Male | Always | Always | 0 | Middle | cT1N0M0 | ⅡB | G2 | R0 | pT1bN1M0 |
| 7 | 75 | Female | Never | Never | 0 | Middle | cT3N0M0 | ⅢB | G2 | R0 | pT3N1M0 |
| 8 | 45 | Male | Never | Never | 0 | Middle | cT3N1M0 | ⅢB | G2 | R0 | pT3N2M0 |
| 9 | 44 | Male | Always | Always | 0 | Middle | cT2N1M0 | ⅢB | G2 | R0 | pT2N2M0 |
| 10 | 72 | Male | Always | Never | 0 | Middle | cT2N0M0 | ⅢA | G2 | R0 | pT2N1M0 |
| 11 | 67 | Male | Always | Never | 0 | Middle | cT3N1M0 | ⅢB | G2 | R0 | pT3N1M0 |
| 12 | 67 | Male | Always | Always | 0 | Middle | cT3N0M0 | ⅢB | G2 | R0 | pT3N1M0 |
| 13 | 55 | Male | Never | Never | 0 | Middle | cT3N1M0 | ⅢB | G3 | R0 | pT3N2M0 |
| 14 | 67 | Male | Never | Never | 0 | Middle | cT3N0M0 | ⅢB | G2 | R0 | pT3N1M0 |
| 15 | 52 | Male | Never | Never | 1 | Middle | cT3N1M0 | ⅢB | G1 | R0 | pT3N1M0 |
| 16 | 58 | Female | Never | Never | 1 | Middle | cT1aN0M0 | ⅡB | G2 | R0 | pT1aN1M0 |
| 17 | 74 | Male | Always | Always | 0 | Middle | cT3N0M0 | ⅢB | G2 | R0 | pT3N1M0 |
| 18 | 76 | Male | Never | Never | 1 | Middle | cT2N0M0 | ⅢB | G2 | R0 | pT2N2M0 |
| 19 | 63 | Male | Always | Always | 1 | Middle | cT3N0M0 | ⅢB | G3 | R0 | pT3N2M0 |
| 20 | 77 | Male | Always | Former | 0 | Middle | cT3N0M0 | ⅢB | G2 | R0 | pT3N1M0 |
| 21 | 73 | Male | Never | Always | 0 | Middle | cT3N0M0 | ⅢB | G2 | R0 | pT3N1M0 |
| 22 | 77 | Male | Never | Always | 0 | Middle | cT3N1M0 | ⅢB | G2 | R0 | pT3N2M0 |
| 23 | 55 | Male | Always | Never | 0 | Middle | cT2N0M0 | ⅢA | G2 | R0 | pT2N1M0 |
| 24 | 59 | Male | Always | Always | 0 | Middle | cT2N0M0 | ⅢA | G2 | R0 | pT2N1M0 |
| 25 | 68 | Female | Never | Never | 0 | Up | cT1bN0M0 | ⅡB | G2 | R0 | pT1bN1M0 |
| 26 | 68 | Male | Never | Never | 0 | Middle | cT3N0M0 | ⅢB | G2 | R0 | pT3N1M0 |
| 27 | 71 | Male | Always | Former | 1 | Middle | cT3N1M0 | ⅢB | G2 | R0 | pT3N1M0 |
| 28 | 73 | Female | Never | Never | 0 | Middle | cT2N0M0 | ⅢA | G1 | R0 | pT2N1M0 |
| 29 | 52 | Male | Former | Never | 0 | Middle | cT3N0M0 | ⅢB | G3 | R0 | pT3N2M0 |
| 30 | 74 | Male | Never | Never | 0 | Down | cT3N1M0 | ⅢB | G2 | R0 | pT3N2M0 |

Supplementary Figure 1


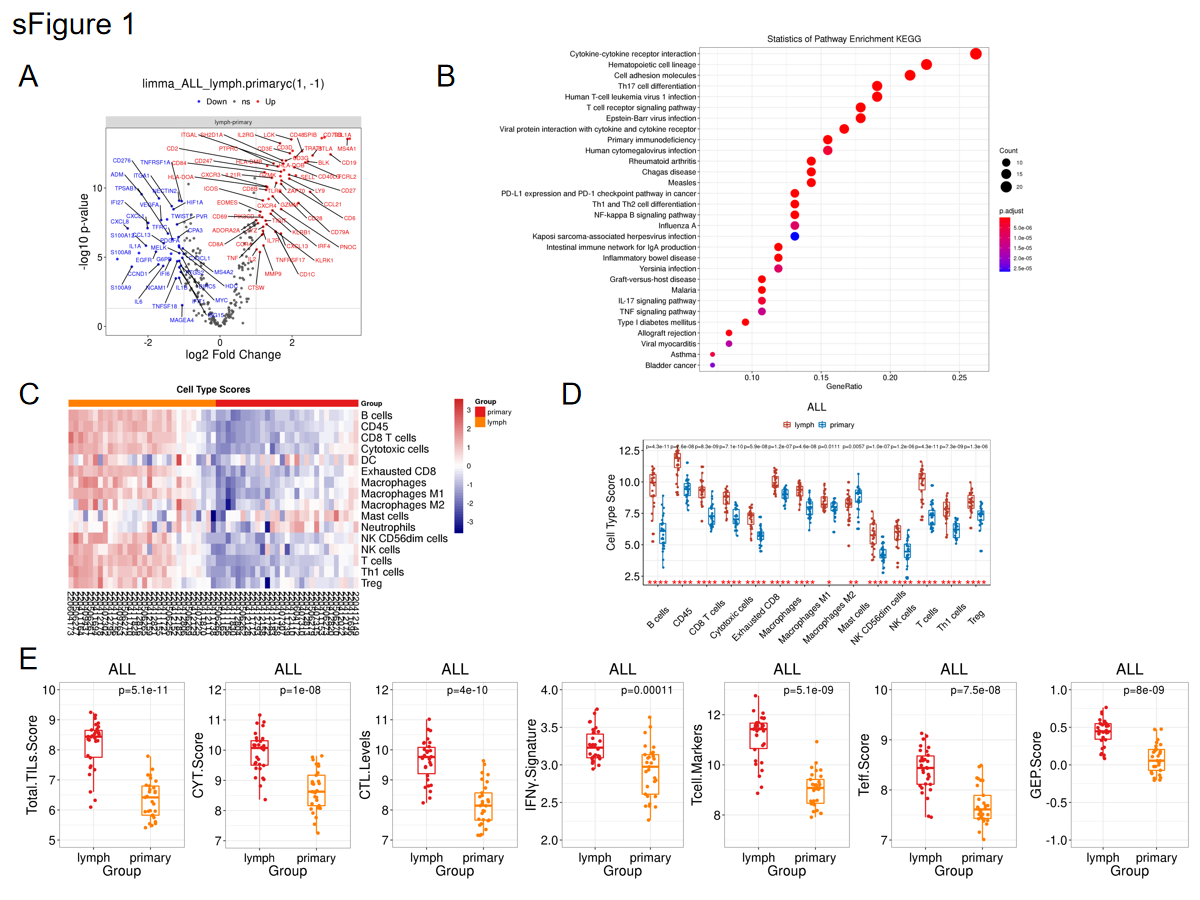


Supplementary Figure 2


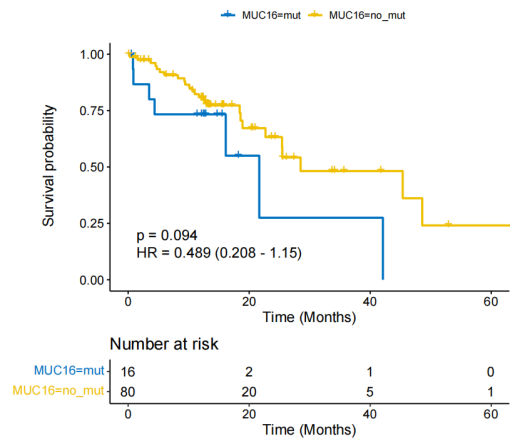

Supplement: Supplementary file 1 — Data S1. [file CAM4-13-e7228-s001.docx]
